# Tau in Alzheimer's disease: Shaping the future patient journey

**DOI:** 10.1002/alz70858_099837

**Published:** 2025-12-24

**Authors:** Douglas W. Scharre

**Affiliations:** ^1^ Ohio State University Wexner Medical Center, Columbus, OH, USA

## Abstract

The introduction of tau‐targeting Disease Modifying Therapies (DMT) to the existing treatment landscape for Alzheimer's disease (AD) holds the promise of enabling a more personalized approach for optimal and timely early‐stage treatment strategies. However, it will require increased healthcare system capacity for the expanded patient influx, utilization of patients and primary care providers (PCP) for early identification of cognitive changes, deciding upon a comprehensive clinical biomarker profile, and careful discussion of potential risks and benefits in coordination with patient preferences. Healthcare systems may need to shift towards decentralized and alternate care models like telehealth. At home or PCP office digital, cognitive self‐assessments monitored over time can improve efficiency of early cognitive impairment identification. PCP can rule out non‐degenerative causes with imaging and labs, then utilize blood‐based biomarkers to identify those individuals to refer to cognitive specialists who utilize a triage process to bypass long wait times. These specialists can determine the appropriate patient for specific monotherapy, combination therapy, or sequential DMT therapy by reviewing eligibility and exclusion recommendations, utilizing specific biomarker (e.g. blood, CSF, PET imaging) profiles, and considering many other factors including patient age, APOE genotype, AD stage, family support, patient preferences, level of risk aversion, burdens of treatments and monitoring (side effects, adherence to requirements), routes of treatment administration (e.g. subcutaneous, intravenous, intrathecal), and the clinician's experience in providing these therapies. This information is critically important to make personalized evidenced‐based treatment decisions for the patient. Specialized patient tracking systems can be designed to monitor treatment progress, adverse events, scheduling of safety procedures, recommended pausing or stopping criteria, change in cognitive, functional, and biomarker status, and to communicate efficiently any adjustments in workflows, monitoring, and patient tracking to all parties. At multiple time points, patients and their proxies must be able to reassess their treatment plan based on ever changing circumstances. Biomarkers will shift values post‐treatments which necessitates new frameworks for diagnosis, staging, and treatment. Use of tau‐directed drugs in the treatment landscape should be data‐driven following clinical and real‐world investigations, with the goal of optimizing patient functioning and independence.

